# A spatio-temporal analysis of dengue spread in a Brazilian dry climate region

**DOI:** 10.1038/s41598-021-91306-z

**Published:** 2021-06-04

**Authors:** Aloísio S. Nascimento Filho, Thiago B. Murari, Paulo Ferreira, Hugo Saba, Marcelo A. Moret

**Affiliations:** 1 Centro Universitário Senai Cimatec – Salvador, Bahia, Brazil; 2 VALORIZA - Research Center for Endogenous Resources Valorization, Portalegre, Portugal; 3 Instituto Politécnico de Portalegre, Portalegre, Portugal; 4 CEFAGE-UE, IIFA, Universidade de Ev́ora, Ev́ora, Portugal; 5 Universidade do Estado da Bahia, Salvador, BA Brazil

**Keywords:** Socioeconomic scenarios, Health policy, Viral infection

## Abstract

We investigated the relation between the spread, time scale, and spatial arrangement of dengue in Bahia, a Brazilian dry climate region, for the period 2000 to 2009. The degree of cross-correlation is calculated for 15 economic regions. We propose a multiscale statistical analysis to datasets of dengue cases in order to verify the effect of infection dispersal on the economic regions from the metropolitan region of Salvador. Our empirical results support a significant and persistent cross-correlation between most regions, reinforcing the idea that economic regions or climatic conditions are non-statistically significant in the spread of dengue in the State of Bahia. Our main contribution lies in the cross-correlation results revealing multiple aspects related to the propagation of dengue in dry climate regions.

## Introduction

Dengue, Zika virus and Chikungunya are caused by arboviruses that are transmitted in urban centers by the same arthropod vector, Aedes aegypti mosquitoes (AA). Once infected, the mosquito can transmit the virus during its whole lifetime. However, the course of an infection differs between people. The transmission of these arboviruses is complex, involving the various aspects of the vectors’ behavior as well as that of the infected humans^1–8^. Studying the tracking and history of a disease has been a way to understand its behavior^9^. We believe that evaluating temporal interactions of case records may reveal relevant clues about the disease spread process, mainly in large areas. For instance, large cities can provide favorable conditions for arbovirus transmission due to the easy circulation of infected people.

Spatial analysis has been able to construct scenarios and identify health impacts^10^. Studies involving spatial and temporal disease have tried to identify patterns of the dengue infection. For instance, spatial and temporal patterns of dengue infection were studied in two cities in Taiwan by applying cluster analysis and two distinct diffusion behaviors were found, a rapid dispersal and a relocation pattern of temporal movement involving clusters^11^. A study involving pattern the dengue patter and hemorrhagic dengue incidence in several districts was applied in the Songkhla municipality in Thailand. The findings revealed that the incidence was strongly associated with the percentages of shop-houses, brick-built houses and houses with poor garbage disposal^12^.

**Figure 1:**
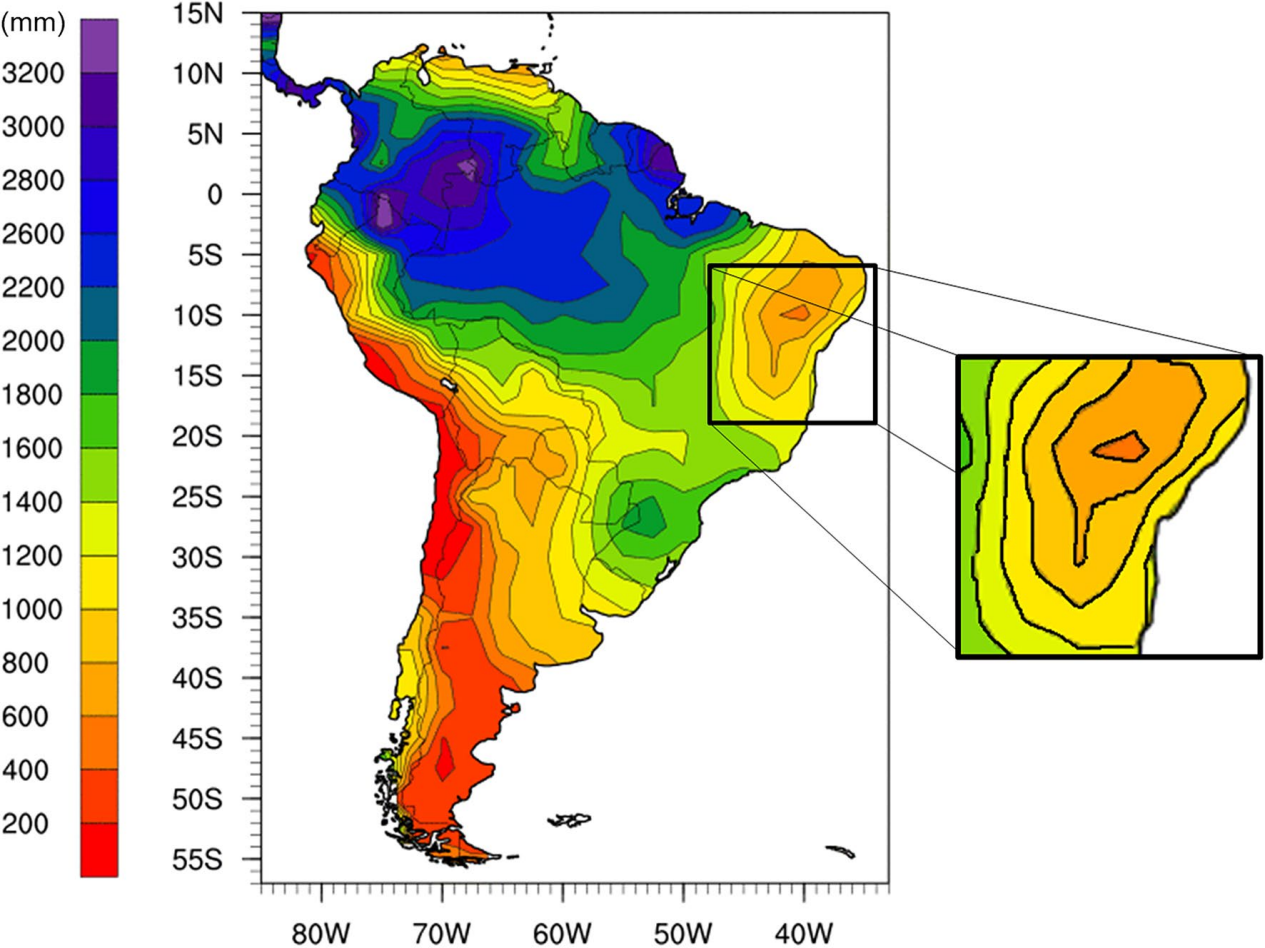
Annual total precipitation in Bahia. According to the Physical Science Division of U.S. department of commerce between 1976 and 2009 this was less than 1000 mm. Figure adapted from^15^.

Brazil has natural features that make it vulnerable to arboviral diseases. In recent years, Zika virus and Chikungunya joined dengue as relevant Brazilian public health problems. As data on Zika virus and Chikungunya data are still scarce, using dengue data may provide important clues about their spread patter. Bahia is the fourth largest region of Brazilian in terms of population, with 14, 016, 906 inhabitants. Covering 564,732 km^2^, it has an extensive road network interconnecting all municipalities in the state^13^. Besides, Bahia has specific climatic conditions such as low precipitation, as shown in Fig. 1. The transportation network in Bahia consist of 22 federal highways and 11 state highways covering all 417 municipalities^14^. As AA is present in 99.5% of municipalities in Bahia, combating the spread of arboviruses is a challenge for public health.

With^1^ high levels of precipitation and a temperature suitable for dengue transmission, there is a strong association with elevated dengue risk, although low precipitation was not found to limit transmission greatly. A study measuring the existence of a spatial correlation between socioeconomic, demographic and environmental variables and the incidence of dengue in a medium-sized city in Brazil, found spatial dependence of the incidence of dengue, socioeconomic factors and the organization of urban space^16^. For insentience, the relevance in the diffusion of dengue across the state of Bahia-Brazil was observed by applying correlations between occurrences of reported cases of dengue among municipalities^9,14,17^. Another study evaluated whether the spread of dengue fever can be explained by differences in regional economies, concluding that this disease was not influenced by economic aspects or regional arrangement, and also suggesting that the disease’s AA vector has adapted to all economic regions^18^.

The diffusion of disease may be observed as a complex system. Thus, Self-Organized Criticality (SOC)^9,19^, fractal behaviors^18,20^, non-linear systems^8^ among other features could be useful in the analysis of spread of diseases. In relation to dengue, the transportation presents correlations among criticality, physical means of propagation, and distribution of dengue cases^17,21^. The fractal behavior presents correlation with SOC exponents^20^. In this sense, analyzing fluctuations allows us to verify features of the evolution of dengue that are not evident when we use traditional methods.

As there is no effective chemical and biological vector control, and the fact that no vaccine against dengue, Zika virus and Chikungunya are not yet available, the hypothesis of the pathways and speed of arbovirus circulation in Bahia-Brazil may be relevant factors to interrupt the spread of arboviruses. As time series of dengue are available, we propose to evaluate the speed and dynamics of the spread of the disease. To do so, we verify the pathways of dengue by applying the cross-correlation *DCCA* statistical method to dengue cases Bahia-Brazil between 2000 and 2009.

## Results

**Figure 2:**
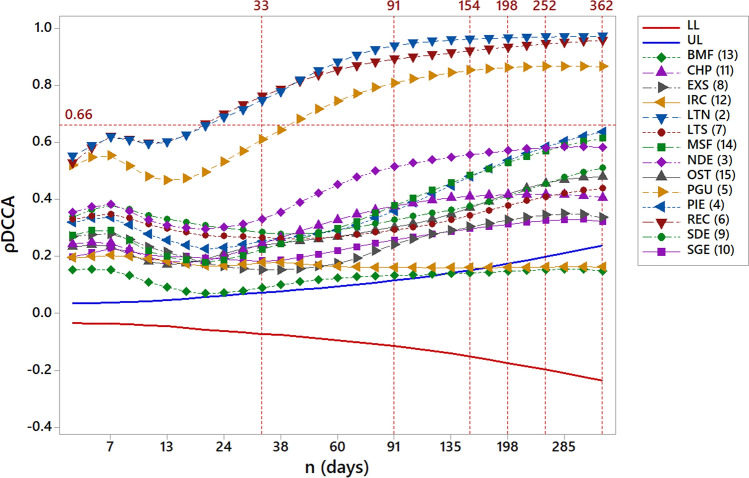
Cross-correlation results for 29 different time scales. The values for $\rho _{DCCA}$ between RMS and the nearby ER presented a strong, significant correlation. LTN, REC and PGU stood out by exceeding the correlation level of 0.66, for the scale of 33 days, remaining strong for all subsequent time scales. LL and UL represent the low and upper limits respectively, and blue line and red line are the significant level values calculated with confidence intervals equal to 95% through the time scales (weeks)^23^. The labels as follows are: economic regions in Bahia, Brazil. The metropolitan region of Salvador (RMS), Litoral Norte (LTN), Nordeste (NDE), Piemonte da Diamantina(PIE), Paraguaçu (PGU), Recôncavo (REC), Litoral Sul (LTS), Extremo Sul (EXS), Sudoeste (SDE), Serra Geral (SGE), Chapada Diamantina (CHP), Irecê (IRC), Baixo Médio São Francisco (BMF), Médio São Francisco (MSF), Oeste (OST).The division was extracted from^24^.

We calculated 105 pairs for 29 different time scales, considering boxes from 4 to 362 days. Figure 2 shows the cross-correlations between RMS with all ER. The following regions presented a strong cross-correlation coefficient after 33 days: LTN, REC and PGU. A cross-correlation is considered strong when the $\rho DCCA$ is greater than 0.66^22^. All the other regions did not show a strong cross-correlation coefficient, but they are statistically significant, except IRC and BMF, which become non-significant after 154 days.

**Figure 3:**
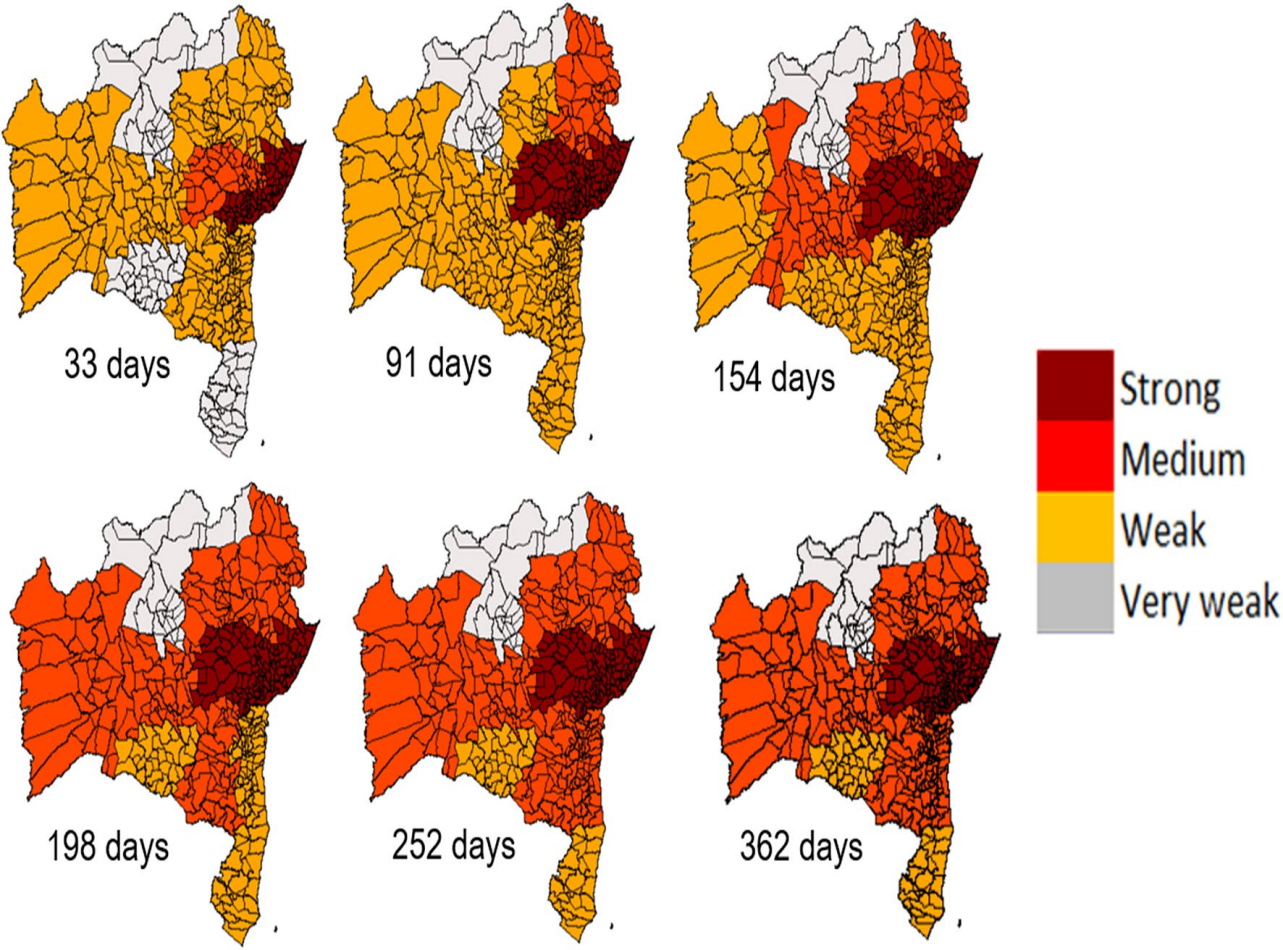
Correlation degree between RMS and all economic regions in Bahia-Brazil for different time scales. The value of the $\rho _{DCCA}$ between RMS and all ER presented a significant correlation. This was calculated for six scale days, 33, 91, 154, 198, 252 and 362 days. The $\rho _{DCCA}$’s ranges were associated with colors (Strong: $\rho _{DCCA} > 0.66$, Medium: $0.4 < \rho _{DCCA} \le 0.66$, Weak: $0.2 < \rho _{DCCA}\le 0.4$, and Very weak: $\rho _{DCCA}\le 0.2$) to assist understanding.

The pathways of the dengue spread are shown in Fig. 3. For better visualization of this spread, we proposed a classification for the coefficient degree, as follows: Strong: $\rho _{DCCA} > 0.66$, Medium: $0.4 < \rho _{DCCA} \le 0.66$, Weak: $0.2 < \rho _{DCCA}\le 0.4$, and Very weak: $\rho _{DCCA}\le 0.2$. This shows the continued strong correlation for ER around RMS borders for any time scale. Between 154 and 254 days, there is a weak to medium coefficient movement in almost all ER of Bahia that do not border with RMS, except for EXS, SGE, BMF and IRC. The spread remains unchanged after 254 days. This behavior may suggest that the spread directly associated with the distance between municipalities.

This dengue spread behavior seems to be related to the direct links between RMS and other ER, represented in Fig. 6. Direct links are located mostly in the border regions of RMS. The exceptions are BMF and IRC, which have direct links but are in the very weak range of the coefficient degree. These two ER are within the area with the lowest precipitation in the region analyzed, as can be observed in Fig. 1.

## Discussion

The multiscale statistical analysis was applied to the datasets of dengue cases to determinate the effect of infection dispersal on the economic regions, starting in the metropolitan region of Salvador. It is the first time the $\rho _{DCCA}$ method has been used to assess the spread of dengue in a dry region of the world. The interaction of distinct economic units was observed the in Bahia from the metropolitan region of Salvador, with the apparent interconnection having the capacity to form the transmission chain, and hence the spatio-temporal spread of the virus.

The considerable increase of degree coefficient allowed determinate of the time scale dynamic of the spread of dengue in a dry region of Brazil, where in the begging the $\rho _{DCCA}$ coefficient becomes strong after 33 days for economic regions bordering RMS, as shown in Fig. 3. The co-movements provided by $\rho _{DCCA}$ has the advantage of setting aside local and regional details, such as economic aggregates, areas, and distances, giving an opportunity to evaluate the dengue propagation pathways and its dynamic.

The empirical results support a significant and persistent cross-correlation between most economic regions. These co-movements between economic regions and the metropolitan region of Salvador do not seem to be statistically impacted by the state’s climatic conditions. The central region of the map, which is extremely dry (Fig. 1) and where the region number 5 is found, is strongly correlated with the metropolitan region (1) after 91 days.

Likewise, almost all economic regions have a medium or strong cross correlation with the metropolitan region of Salvador in the period of one year. Thus, we can conclude that the economic division defined by law No. 6,349 of the state of Bahia is not a significant barrier to the spread of dengue across the state from the metropolitan region of Salvador.

The scope of the study is limited to the state of Bahia, Brazil. Our main contribution lies in the co-movement results, revealing multiple aspects related to the propagation of dengue in these dry climate regions. Finally, the model has some level of uncertainty, an inherent limitation of the model assumption as a large time series. Other analysis, such as assessing municipalities individually and the impacts on neighborhood co-movements, may be easily implemented. Factors such as the spatial distribution of the transport network and the movement of people between cities can also be considered to analyze co-movements.

## Methods

**Figure 4:**
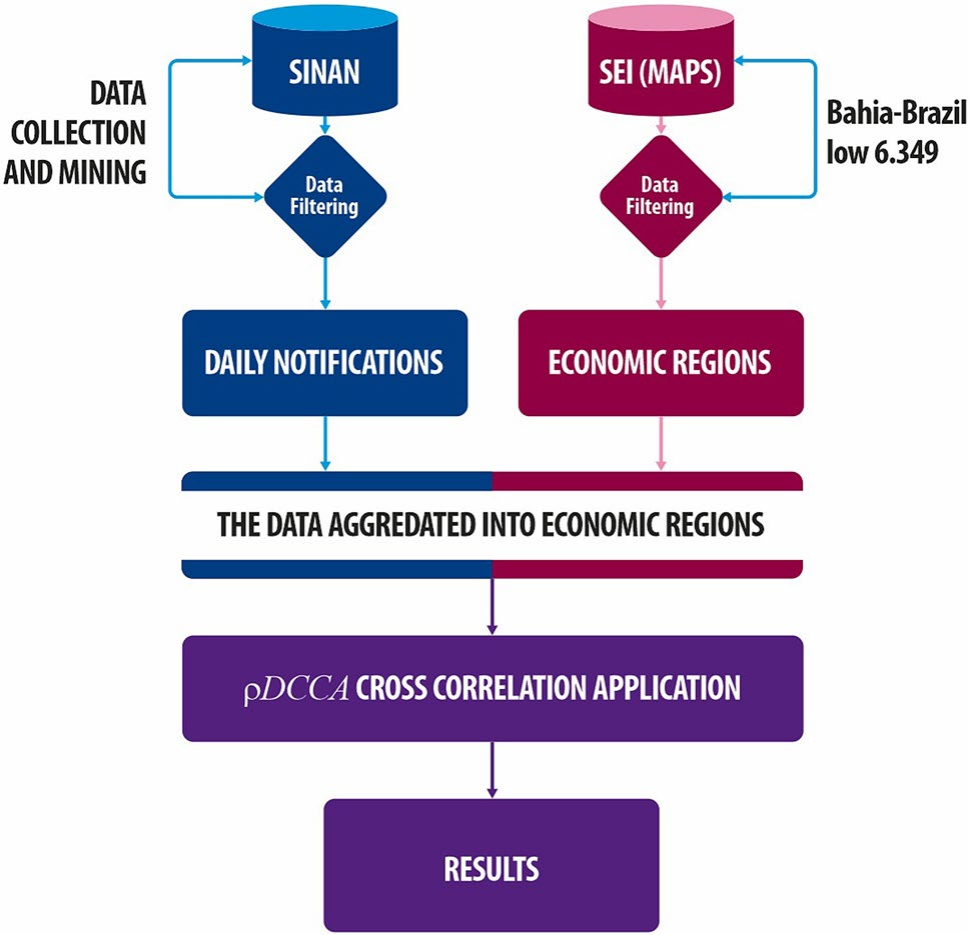
Daily records of dengue cases are available by Brazilian Diseases Notification System databases (SINAN)^25^. The economic arrangement proposed by the Superintendence of Economic and Social Studies State of Bahia-Brazil (SEI-BA)^24^. Among several arrangements, the SEI-BA provides the Economic Regions (ER) which is supported by Bahia’s low # 6.349^24^. The $\rho _{DCCA}$ cross correlation analysis method^26^ was applied at SINAN filtered data aggregated by ER.

**Figure 5:**
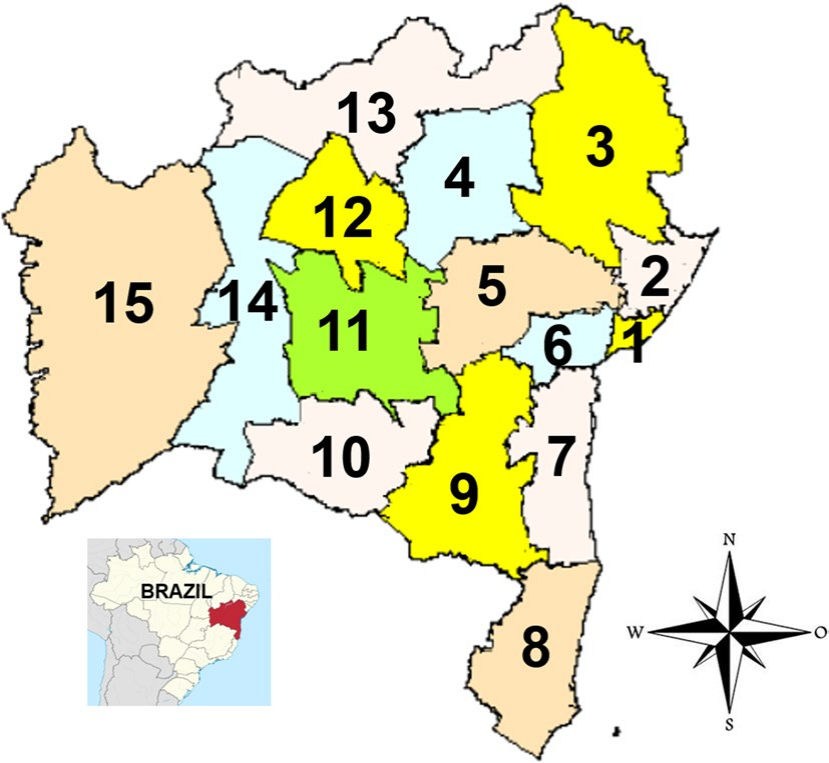
Economic regions in Bahia, Brazil. 1-The metropolitan region of Salvador (RMS), 2-Litoral Norte (LTN), 3-Nordeste (NDE), 4-Piemonte da Diamantina(PIE), 5-Paraguaçu (PGU), 6-Recôncavo (REC), 7-Litoral Sul (LTS), 8- Extremo Sul (EXS), 9-Sudoeste (SDE), 10-Serra Geral (SGE), 11-Chapada Diamantina (CHP), 12-Irecê (IRC), 13-Baixo Médio São Francisco (BMF), 14-Médio São Francisco (MSF), 15-Oeste (OST). The map was extracted from^24^.

For better understanding the methodology applied, the schema of data analysis was provided in Fig. 4, where the daily records of dengue cases in Bahia were collected between 2000 and 2009 from the Brazilian Diseases Notification System databases (SINAN) from the Brazilian health ministry^25^. We note that the database used in this paper does not identify individuals and they were notified in urban areas. The dengue records were organized into Economic regions, an arrangement used by the Superintendence of Economic and Social Studies for the State of Bahia-Brazil (SEI-BA) and created by Bahia’s low 6,349, which divides the State into 15 economic regions (see Table 1)^24^. The data on dengue cases data were aggregated in each economic region respectively. Figure 5 shows the map to assist understanding.

**Table 1:** Economic Regions: simbols.

Id	Symbol	Economic region	Quantity of cities
1	RMS	The metropolitan region of Salvador	10
2	LTN	Litoral Norte	20
3	NDE	Nordeste	46
4	PIE	Piemonte da Diamantina	24
5	PGU	Paraguaçu	42
6	REC	Recôncavo	33
7	LTS	Litoral Sul	53
8	EXS	Extremo Sul	21
9	SDE	Sudoeste	39
10	SGE	Serra Geral	29
11	CHP	Chapada Diamantina	33
12	IRC	Irecê	19
13	BMF	Baixo Médio São Francisco	9
14	MSF	Médio São Francisco	16
15	OST	Oeste	23

**Figure 6:**
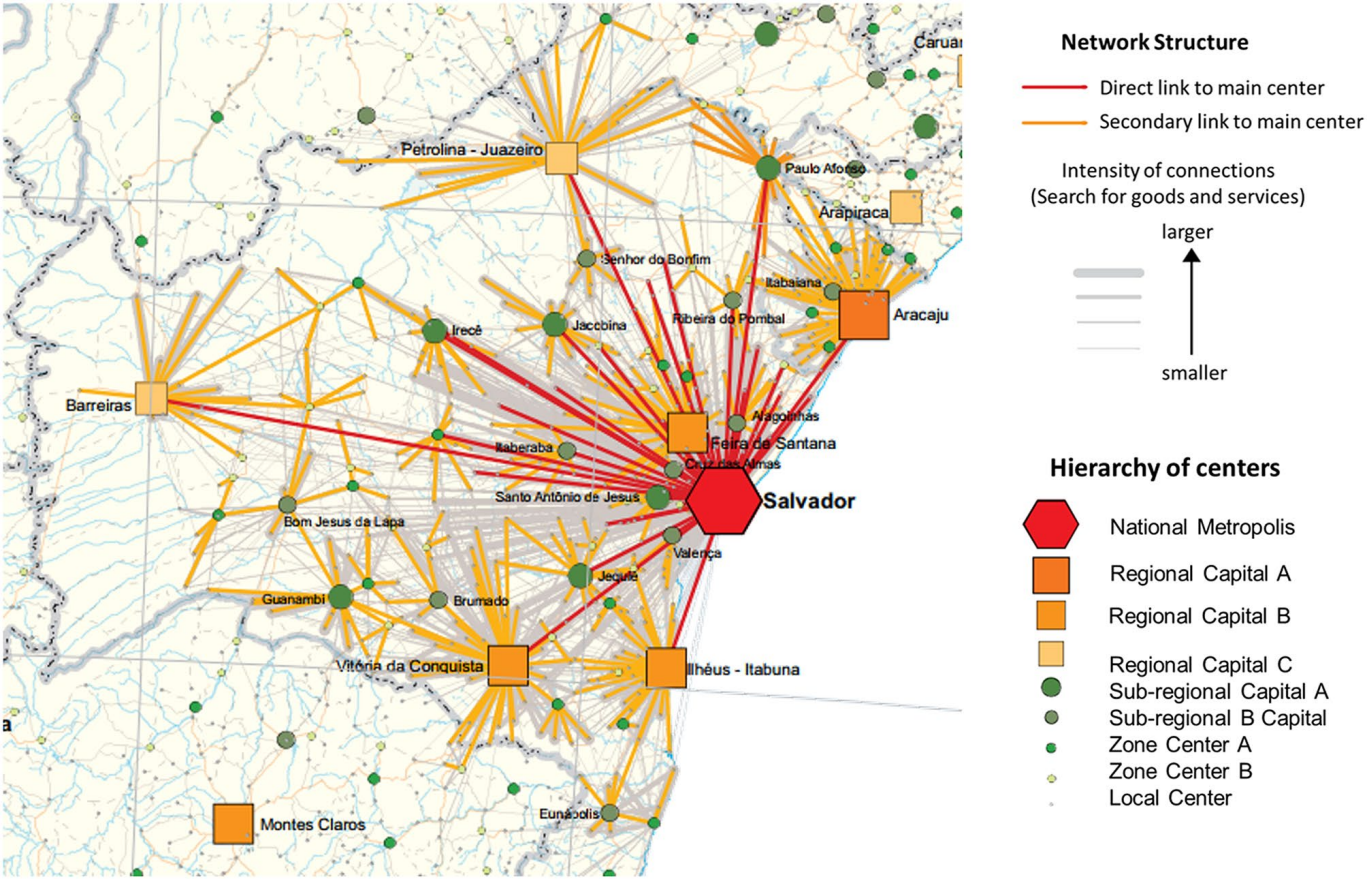
Salvador capital of Bahia, into the RMS is characterized by include intense connections between cities. It plays a role of territorial management, evaluating levels of centrality of the executive and judiciary at the federal level, and of corporate centrality, as well as having different facilities and services. Figure adapted from^13^.

The most important ER (RMS) was chosen in this analysis due to its economic importance and to include the capital of Bahia, Salvador. Furthermore, Salvador is classified as a Regional City of Influence^13^ as showed in Fig. 6. Such cities are characterized by the intensity of the connections between cities cities. They play a role in territorial management, evaluating levels of centrality of the executive and judiciary at the federal level, and of corporate centrality, as well as the presence of different facilities and services.

The assumption of stationarity of random variables is required to perform many statistical inference methods. For instance, when we want to correlate distinct data sets in order to predict their behaviours, the non-stationary in the signals makes the standard Pearson correlation coefficient useless in the this hypothetical evaluation. Thus, it is necessary to use statistical models that develop to accommodate the complexity of the data. The detrended cross-correlation (DCCA) method can estimate the true correlation coefficient between series precisely^27^. This method is robust to contaminated noises, such as daily dengue records, and the DCCA methods have some advantages in correctly quantifying scale-dependent correlations^28^.

The $\rho _{DCCA}$ cross-correlaion coefficient method used is a proposal of^26^, based on the self-affinity theory (fractal object)^29^ and the study of cross-correlation between time series defined by power laws^30^. In this context, there is some recent progress in cross-correlations method families, see^27,31–36^. For the explanation how to calculate the $\rho _{DCCA}$ cross-correlation coefficient, see Supplementary Information, section S1.

In analysis of the results, also considered was the statistical test of^23^ in which the correlation is significant outside the lines representing lower and upper critical values with significance of 95%, for the test of hypothesis H0:$\rho _{DCCA} = 0$ and H1: $\rho _{DCCA} \ne 0$.

## Supplementary Information

Supplementary Information.
