# Supplementary material for: A spatio-temporal analysis of dengue spread in a Brazilian dry climate region
Source: Sci Rep. 2021 Jun 4;11:11892. doi: 10.1038/s41598-021-91306-z (PMC8178350; doi:10.1038/s41598-021-91306-z)
Supplement: Supplementary file 1 — Supplementary Information. [file 41598_2021_91306_MOESM1_ESM.pdf]

Supplementary Information -S1

The ρDCCA method[16]is based on the ratio between the detrended covariance function F_{xy}^2 of the Detrended Cross-Correlation Analysis method[20] and the variance function without trend $F_{zz}(with\ z = x\ or\ z = y)$ of the Detrended Fluctuation Analysis method (DFA)[19]. It is shown in five steps below:

- **Step I:** Starting from two different time series, x_i and y_i, with $i = 1, 2, \ldots, N$ (time series length). From the original time series two new integrated time series are obtained (Eq.1):

$$X_k = \sum_{i=1}^{k} [x_i - \bar{x}]\ and\ Y_k = \sum_{i=1}^{k} [x_i - \bar{y}] \tag{1}$$

where $\bar{x}$and $\bar{y}$represent the mean value of each time series, and $k = 1, \ldots, N$.

Step II: We divide these two integrated time series, $X_k$and Y_k, into $(N - n)$ overlapping boxes of equal length n, where $4 \leq n \leq \frac{N}{4}$, as shown in Figures 1 and 2. The original data present a total N of dengue case observations equal to 3,404 days, with a minimum scale equal to 4 days and a maximum scale of $N/4$. As we have 3,404 observations, the maximum would be 851 boxes. The scale of growth of each time series follows the DFA algorithm[19]. On The Research Resource for Complex Physiologic Signals (PhysioNet) website the DFA scale method is available, (PhysioNet - software projects, https://physionet.org/content/dfa).

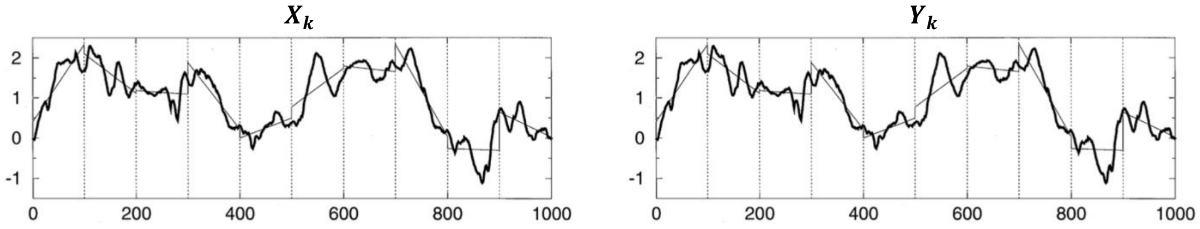

Figure 1: The illustration of the division of the two integrated signals $X_k$and Y_k.

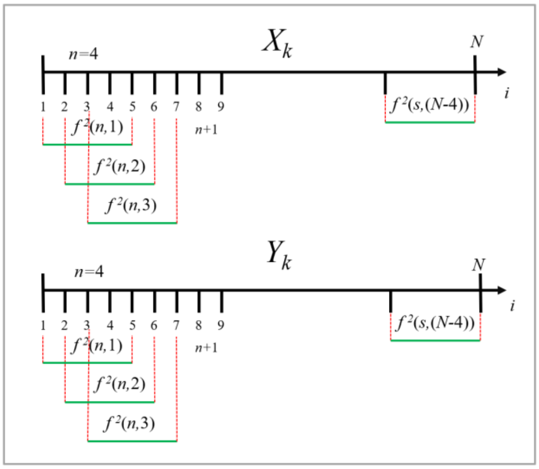

Figure 2: Division of the integrated signals $X_k$and Y_k into $(N - n)$ boxes overlapping boxes n size $n = 4$, each containing $n + 1$ values.

- Step III: After this step, the polynomials (order one here) that best adjust the points (by least-squares fit) in each box of each series, $\tilde{R}_{K,i}$ and $\tilde{R}'_{K,i}$, are calculated and therefore the covariance of the residuals in each box (length n):

$$f^2DCCA(n,i) = \frac{1}{n+1}\sum_{i}^{i+n}(R_k - \tilde{R}_{K,i})(R'_k - \tilde{R}'_{K,i}) \tag{2}$$

- Step IV: The mean over all $(N - n)$ overlapping boxes is calculated to obtain the detrended covariance function:

$$F_{xy}^2(n) \equiv \frac{1}{N+n}\sum_{i=1}^{N-n} f^2{}_{DCCA}(n,i) \tag{3}$$

- Step V: Finally, $\rho DCCA$ can be calculated by:

$$\rho DCCA(n) = \frac{F_{xy}^2}{F_{xx}(n)F_{yy}(n)} \tag{4}$$

According to [1] the functions $F_{xx}$and $F_{yy}$are respectively the root mean square fluctuation of each time series x_i and $\{y_i$ separately. $\rho DCCA(n)$ ranges from: $-1 \leq \rho DCCA(n) \leq 1$. When $\rho DCCA(n) = 1$ this means a perfect cross-correlation, $\rho DCCA(n) = 0$ there is no cross-correlation, and $\rho DCCA(n) = -1$ means a perfect anti cross-correlation.
